# Profuse Sweating and Hot Flashes: An Unusual Presentation of Post-Dengue Fatigue Syndrome

**DOI:** 10.7759/cureus.10024

**Published:** 2020-08-25

**Authors:** Maheswaran Umakanth, Ethayakumar Narayanasami, Navaneethakrishnan Suganthan

**Affiliations:** 1 Clinical Medicine, Teaching Hospital, Batticaloa, LKA; 2 Medicine, Base Hospital, Batticaloa, LKA; 3 Medicine, University of Jaffna, Jaffna, LKA; 4 University Medical Unit, Teaching Hospital, Jaffna, LKA

**Keywords:** post dengue fatigue syndrome, hot flashes and profuse sweating

## Abstract

The dengue virus is a mosquito-borne flavivirus that causes dengue fever (DF), dengue hemorrhagic fever, and dengue shock syndrome. DF is characterized by fever, headache, arthralgia, retro-orbital pain, and skin rashes. However, some patients with DF develop post-dengue fatigue syndrome (PDFS) following their initial febrile episode. Fatigue is common during the febrile episode of DF; however, PDFS is defined as the presence of a stubborn sense of tiredness that results in a decreased capacity for physical and mental work. PDFS carries a spectrum of musculoskeletal and neurological features. Following the recovery of DF, vasomotor symptoms such as hot flashes, excess sweating, and mood changes are rare. We present the first reported case of PDFS in a young woman in Sri Lanka.

## Introduction

The dengue virus (DV) is an important arbovirus that infects 390 million people per year, of whom 96 million present with clinical manifestations in nearly 129 countries around the world [1]. However, the burden of DV is more prevalent in Asian countries such as Bangladesh, India, Indonesia, Sri Lanka, Maldives, Nepal, Singapore, and Thailand [1,2]. DV causes a spectrum of clinical features including subclinical infection to dengue fever (DF), dengue hemorrhagic fever (DHF), and the severe and life-threatening condition known as dengue shock syndrome [2].

A spectrum of post-infectious, chronic, and disturbing complications of dengue is known as post-dengue fatigue syndrome (PDFS). Post-infection complications are also seen in other conditions such as infectious mononucleosis, query fever, and Lyme disease [3]. PDFS includes musculoskeletal complications, such as weakness and myalgia, and a variety of neurological complications, such as headache, numbness, and a burning sensation [4]. Fatigue is common during the febrile episode of DF; however, PDFS is defined as the presence of a stubborn sense of tiredness that results in a decreased capacity for physical and mental work. This physical fatigue relates to the subjective sense of tiredness and loss of energy. While other clinical features such as body pain, abdominal pain, poor sleep, morning stiffness, and multiple joint pains have been reported [5], we present the first case from Sri Lanka to include vasomotor symptoms such as hot flashes and profuse sweating.

## Case presentation

A 28-year-old woman presented with a history of fever, headache, and vomiting for the last couple of days. Her platelets started to drop to 10 x 10^9^/L, and she had evidence of free fluid in her abdominal cavity. As her blood dengue-immunoglobulin M antibody and dengue nonstructural protein-1 antigen were positive, her provisional diagnosis was made as DHF. She was in the hospital for seven days and discharged without any complications. Four weeks later, she reported concerns of fever, body pain, and pain in her joints. She also reported experiencing intermittent episodes of profuse sweating, especially in the evening and at night.

Interestingly, she also reported concerns of hot flashes and mood changes along with poor sleep, poor concentration, and poor appetite. She sought medical advice from various doctors, including native doctors. Again, she was admitted to the medical ward, and her body temperature was within reference limits. We arranged for blood testing, including lumbar puncture, the results of which were normal. Her menstrual history was uneventful. The provisional diagnosis of PDFS was made. The results of her laboratory blood investigations are presented in Table 1.

**Table 1:** Laboratory investigations in the first and second admissions Abbreviations: Ig, immunoglobulin; ND1, dengue nonstructural protein-1; Q, query; CSF, cerebrospinal fluid

Investigations	First Admission	Second Admission
Full blood count (10 x 10^9^/L)	3	6
Platelets (10 x 10^9^/L)	10	250
Aspartate aminotransferase (U/L)	150	35
Alanine aminotransferase (U/L)	125	40
Dengue IgM antibody	Positive	-
Dengue IgG antibody	Positive	-
Dengue ND1 antigen	Positive	-
Erythrocyte Sedimentation rate (mm/h)	12	06
Fasting blood glucose (mg/dL)	110	105
Blood culture	Negative	Negative
Monospot test	Negative	Negative
Serology for Q fever	Negative	
Lumbar puncture CSF analysis		Normal

## Discussion

Dengue infection causes a variety of presentations ranging from simple self-limiting fever to multiple organ involvement, including appendicitis, meningitis, rheumatoid-like arthritis, parotitis, and myocarditis [6]. However, some patients with DF develop PDFS after their initial febrile period. Fatigue occurred in approximately 25% of hospitalized DF patients given DF’s complex musculoskeletal and neurological features [7]. However, the exact mechanism for PDFS is not known. The muscular symptoms mainly consist of weakness with or without pain with low levels of exertion. However, neurological features vary, such as headache, blurred vision, excess sweating, poor sleep, excess sleep, acute disseminated encephalomyelitis, encephalopathy, encephalitis, transverse myelitis, Guillain-Barre syndrome, and numbness in the extremities. The degree of fatigue is difficult to calculate; however, a 50% reduction in functional level compared with pre-dengue illness functioning is considered fatigue syndrome [8]. While fatigue during and after DF is common, PDFS is an entirely different entity that follows the initial recovery of acute DF.

During the dengue outbreak, several rare complications were encountered in our clinical practice. However, the incidence of PDFS was lower than the fatigue associated with other viral infections such as infectious mononucleosis [9]. Most of the PDFS patients report muscle pain, tiredness, mental exhaustion, sleep disturbance, and sweating. The immune response to the virus, lactic acidosis, and cytokines may be involved in the mechanism of PDFS [9].

Post-dengue neurological features are well-known complications such as Parkinson-like symptoms, cerebellar ataxia, and plexopathy [10]. However, vasomotor symptoms such as excess sweating, hot flashes, and mood changes occurring months after DF are reported have not yet been reported in Sri Lanka before this case. Our patient was treated with antidepressants; her symptoms gradually improved and completely resolved four months after initiation of the treatment.

## Conclusions

DF is usually a self-limiting illness of low mortality, which can be asymptomatic or present in a spectrum of organ involvement. Uncomplicated DF can become complicated by the development of PDFS. PDFS is not a new phenomenon but can mislead the general practitioner. This case serves to remind physicians that meticulous history taking is of paramount importance for optimal patient outcomes.
